# Supplementary material for: Spatium: A Protein Language Foundation Model for Spatial Proteomics
Source: bioRxiv. 2026 Jul 26:2026.07.23.740264. Preprint. [Version 1] doi: 10.64898/2026.07.23.740264 (PMC13419744; doi:10.64898/2026.07.23.740264)
Supplement: Supplement 1 [file media-1.zip › Supplementary Material.docx]

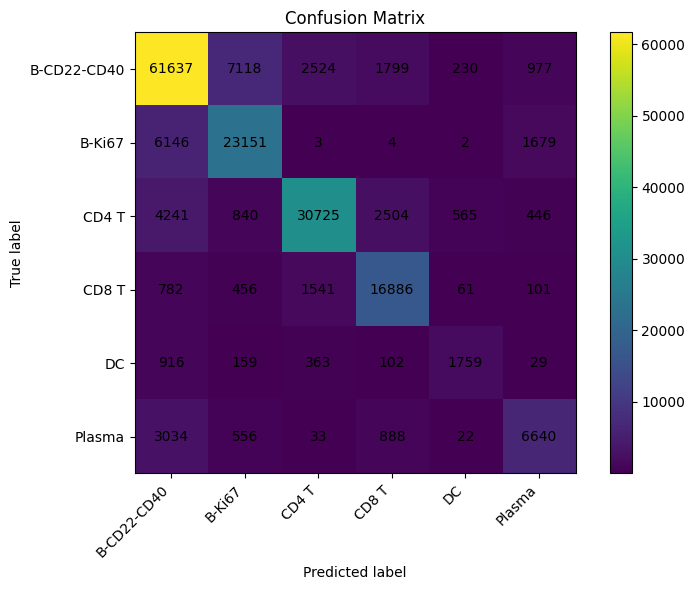


**Fig. 1 Confusion matrix of scRNA -Spatial Proteomics label transfer**.


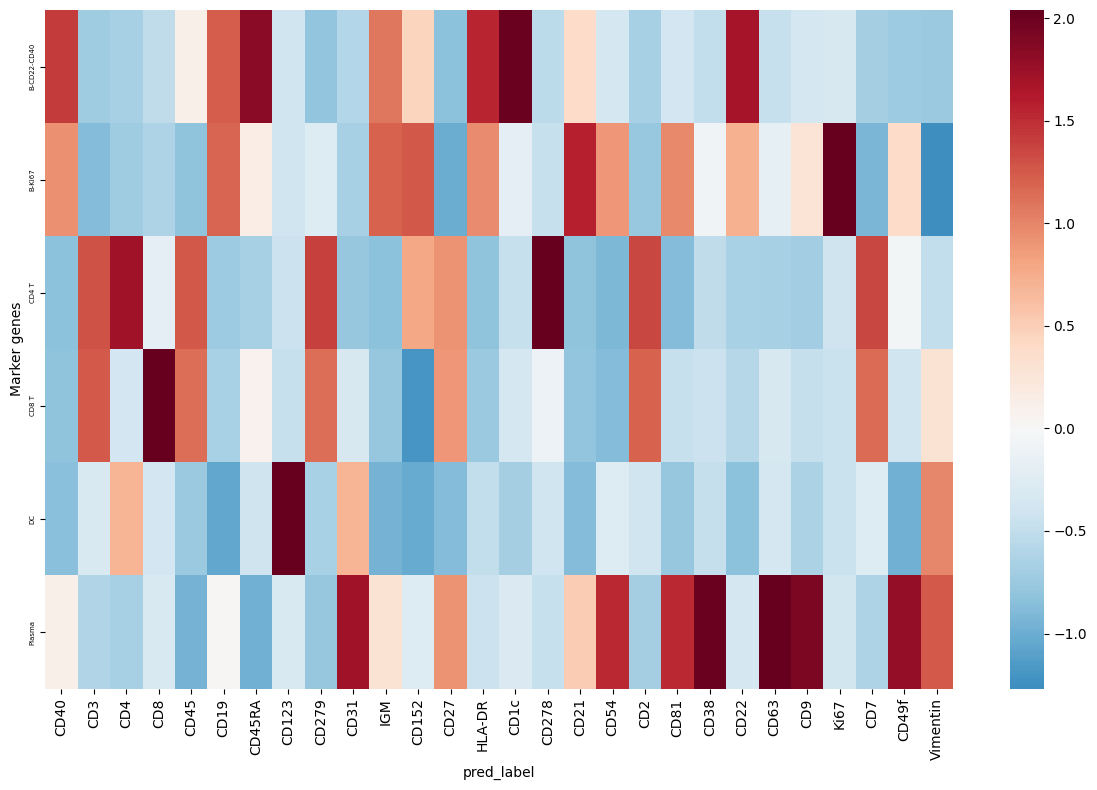


**Fig. 2 Marker distribution in all cell types.**
