# Supplementary figures and images for: Spatium: A Protein Language Foundation Model for Spatial Proteomics
Source: bioRxiv. 2026 Jul 26:2026.07.23.740264. Preprint. [Version 1] doi: 10.64898/2026.07.23.740264 (PMC13419744; doi:10.64898/2026.07.23.740264)

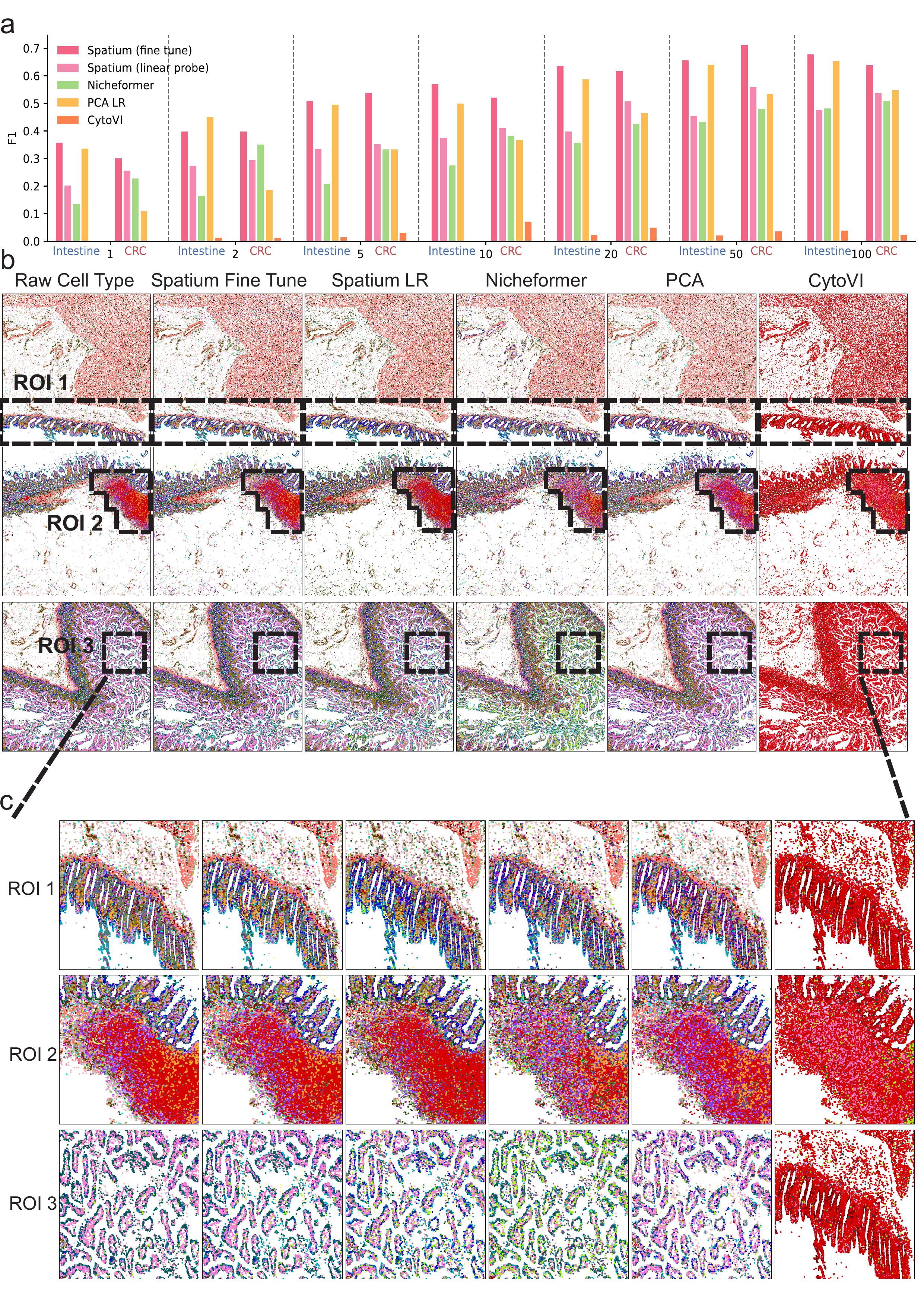

Supplement: Supplement 1 [file media-1.zip › Extended Data Fig 1.png]

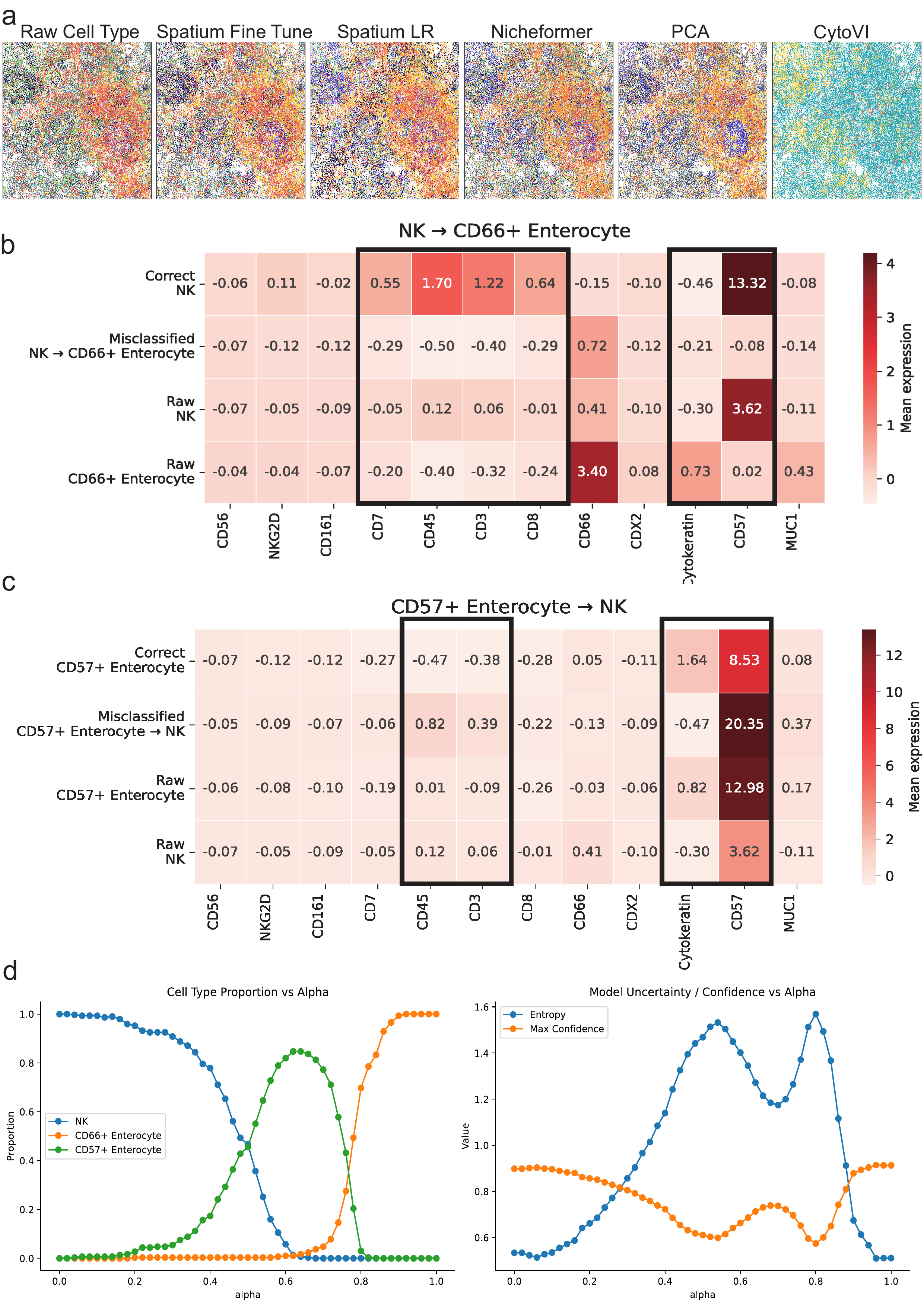

Supplement: Supplement 1 [file media-1.zip › Extended Data Fig 2.png]

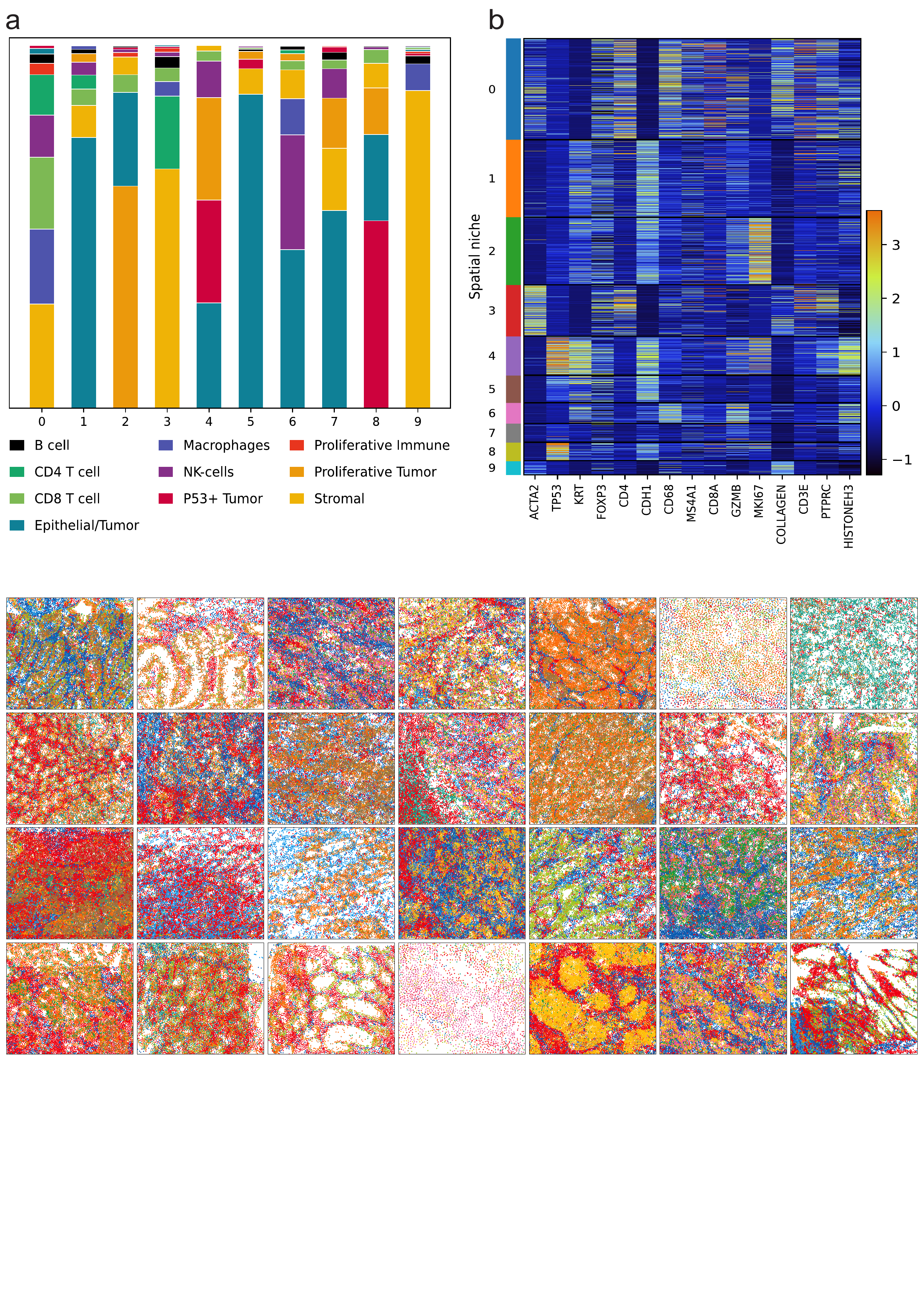

Supplement: Supplement 1 [file media-1.zip › Extended Data Fig 3.png]

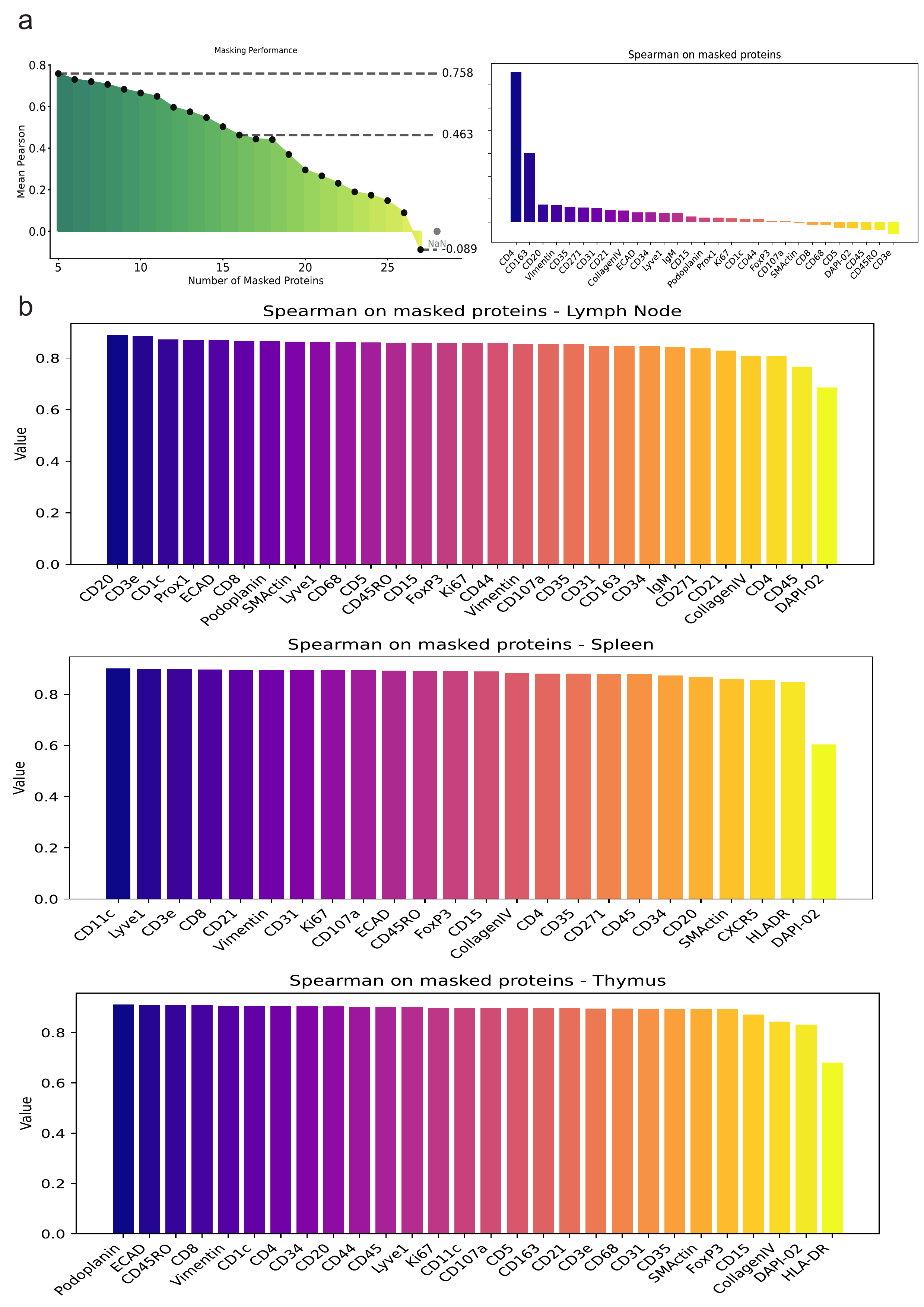

Supplement: Supplement 1 [file media-1.zip › Extended Data Fig 4.png]

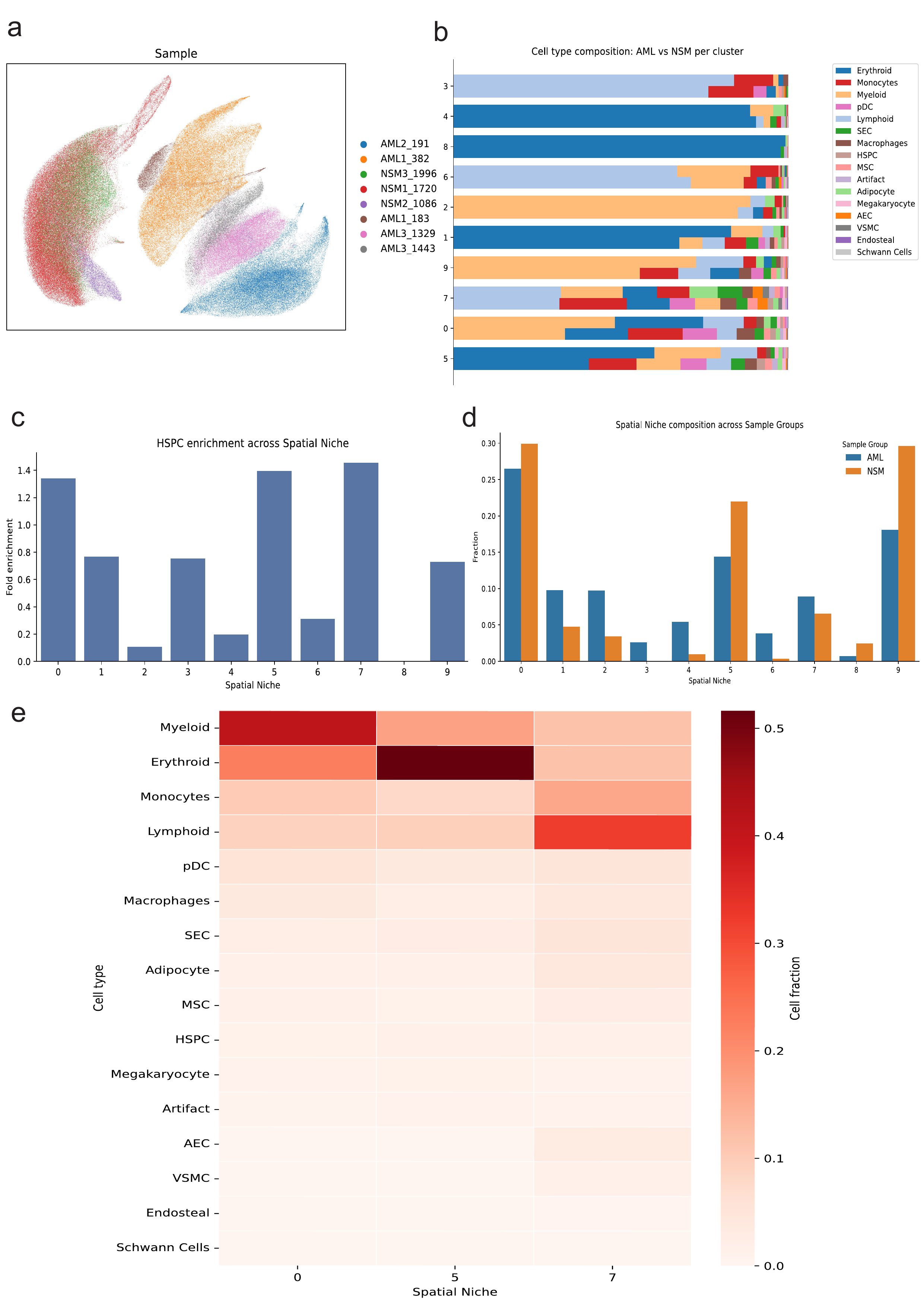

Supplement: Supplement 1 [file media-1.zip › Extended Data Fig 5.png]
